# Host Genetics and Antiviral Immune Responses in Adult Patients With Multisystem Inflammatory Syndrome

**DOI:** 10.3389/fimmu.2021.718744

**Published:** 2021-08-31

**Authors:** Andreas Ronit, Sofie E. Jørgensen, Casper Roed, Robert Eriksson, Ulrik W. Iepsen, Ronni R. Plovsing, Merete Storgaard, Finn Gustafsson, Ann-Brit E. Hansen, Trine H. Mogensen

**Affiliations:** ^1^Department of Infectious Diseases 144, Hvidovre Hospital, University of Copenhagen, Hvidovre, Denmark; ^2^Department of Infectious Diseases, Aarhus University Hospital, Aarhus, Denmark; ^3^Department of Biomedicine, Aarhus University, Aarhus, Denmark; ^4^Department of Infectious Diseases 8632, Rigshospitalet, University of Copenhagen, Copenhagen, Denmark; ^5^Department of Disease Systems Biology, Novo Nordisk Foundation Center for Protein Research, University of Copenhagen, Copenhagen, Denmark; ^6^Department of Anaesthesiology and Intensive Care, Hvidovre Hospital, University of Copenhagen, Hvidovre, Denmark; ^7^Department of Clinical Medicine, University of Copenhagen, Copenhagen, Denmark; ^8^Department of Cardiology and Clinical Medicine, Rigshospitalet, University of Copenhagen, Copenhagen, Denmark

**Keywords:** coronavirus disease 2019, multisystem inflammatory syndrome, SARS-CoV-2, whole exome sequencing, interferon

## Abstract

COVID-19 associated multisystem inflammatory syndrome (MIS) is a rare condition mostly affecting children but also adults (MIS-A). Although severe systemic inflammation and multiorgan dysfunction are hallmarks of the syndrome, the underlying pathogenesis is unclear. We aimed to provide novel immunological and genetic descriptions of MIS-A patients. Cytokine responses (IL-6, IL-1β, TNFα, CXCL10, type I, II and III interferons) following SARS-CoV-2 infection of peripheral blood mononuclear cells *in vitro* were analyzed as well as antibodies against IFNα and IFNω (by ELISA) in patients and healthy controls. We also performed whole exome sequencing (WES) of patient DNA. A total of five patients (ages 19, 23, 33, 38, 50 years) were included. The patients shared characteristic features, although organ involvement and the time course of disease varied slightly. SARS-CoV-2 *in vitro* infection of patient PBMCs revealed impaired type I and III interferon responses and reduced CXCL10 expression, whereas production of proinflammatory cytokines were less affected, compared to healthy controls. Presence of interferon autoantibodies was not detected. Whole exome sequencing analysis of patient DNA revealed 12 rare potentially disease-causing variants in genes related to autophagy, classical Kawasaki disease, restriction factors and immune responses. In conclusion, we observed an impaired production of type I and III interferons in response to SARS-CoV-2 infection and detected several rare potentially disease-causing gene variants potentially contributing to MIS-A.

## Introduction

Coronavirus disease 2019 (COVID-19) in its severe forms may present as a systemic disease with multi-organ dysfunction, but the most common presentation of critical disease constitutes pneumonia with mono-organ respiratory failure. Viral-host interactions underlying this clinical presentation involve evasion mechanisms and immunopathological events, e.g. viral inhibition of type I interferon (IFN) induction (1) and secretion of pro-inflammatory cytokines (2), and other molecules (3, 4), with imbalanced recruitment of inflammatory cells to the lungs (5). Moreover, the role of genetic susceptibility is increasingly being appreciated, e.g. the discovery of inborn errors of IFN immunity (6, 7), which may in particular explain severe disease in younger and healthy individuals.

A minority of severe acute respiratory syndrome coronavirus 2 (SARS-CoV-2) infected individuals develop an inflammatory syndrome with multiorgan affection, which initially was referred to as a Kawasaki-like disease and was first reported in children and adolescents (8). The syndrome is typically seen 2-6 weeks after infection with SARS-CoV-2 and involve several organ systems with fever and elevated markers of inflammation as hallmarks of the condition (9). Differing names and definitions have been used to describe this syndrome, including multisystem inflammatory syndrome in children (MIS-C) with case definitions published by both the Centers for Disease Control and Prevention (10) and the World Health Organization (11). A similar, yet seemingly more severe, syndrome has been reported in adults and named multisystem inflammatory syndrome in adults (MIS-A). An official MIS-A case definition has not been published, and much remains unknown regarding the clinical spectrum and epidemiology. Accordingly, several thousand MIS-C cases have been reported globally (9, 12), whereas the number of MIS-A cases is much lower counting a limited number of case reports (13).

The pathogenesis underlying MIS-C/A remains enigmatic but may involve aberrant immune responses to SARS-CoV-2, including macrophage hyperactivation, antigen-antibody complex deposition, and formation of autoantibodies (14). Recent studies have identified immunological signatures and features of MIS-C including autoantibodies recognizing endothelial, gastrointestinal, and immune-cells, thus linking autoimmunity to specific tissue pathology (15), as well as specific signatures of alarmins, cytotoxicity, TCR repertoire, and plasmablasts (16) and distinct differences in immune profiles distinguishable between MIS-C as opposed to classical Kawasaki disease (17). However, any genetic basis of this apparent immune dysregulation triggered by SARS-CoV2 infection has not been defined. Moreover, as MIS-C and MIS-A are rare disease manifestations, and potentially overrepresented in certain ethnic groups (9), with a susceptibility locus previously being identified for Kawasaki disease (18), it is plausible that MIS-C/A occurs primarily in genetically predisposed individuals.

We here report a case series of MIS-A patients. To provide a further understanding of the host immune response associated with MIS-C/A we studied inflammatory mediators in response to SARS-CoV-2 infection of patient peripheral blood mononuclear cells (PBMCs) *in vitro* and used whole exome sequencing (WES) to identify possible disease-causing genetic variants.

## Materials and Methods

Patients and healthy controls were included from three different Danish University Hospitals following oral and written informed consent after approval from the Danish National and Regional Committee on Health Ethics (1-10-72-80-20 and H‐2–2009‐131). Adult patients with a clinical diagnosis of MIS-A could be included in the case series it they fulfilled the following criteria: documented SARS-CoV-2 infection, prolonged fever, marked inflammation, evidence of clinically severe illness requiring hospitalization, with multisystem (>2) organ involvement including cardiac involvement and exclusion of other plausible diagnoses (10, 11), and were treated with a MIS-A specific therapy. All clinical work-up was performed at the clinician’s discretion.

Blood sampling for each MIS-A patient was performed at various time points after the first symptoms of MIS-A were noticed: Patient 1 (P1): Two months after; P2: On the second day of symptoms; P3: Three weeks after first symptoms; P4: Two weeks after first symptoms; P5: Five days after first symptoms. Blood sampling for all ICU patients with severe COVID-19 was performed <72 hours after mechanical ventilation was initiated.

### Peripheral Blood Mononuclear Cells Purification and Plasma Collection

PBMCs were purified from heparinized blood by Ficoll density centrifugation and stored in liquid nitrogen. PBMCs were cultured in RPMI with 10% heat-inactivated fetal bovine serum and 1% penicillin–streptomycin upon thawing. Serum was collected from CAT tubes by centrifugation upon coagulation and stored at -80°C until further use.

### Cytokine Response Following SARS-CoV-2 Infection

PBMCs were seeded in 96-well-plates (500.000 per well), rested overnight, and were subsequently infected with SARS-CoV-2, strain FR-4286, at a multiplicity of infection of 0.5 for 24 h. Supernatants were harvested, inactivated with 0.4% Triton-X-100 at a ratio of 1:1 for 30 min and stored at -80°C. Cytokine concentration was subsequently measured in supernatants from SARS-CoV-2 infected PBMCs by U-plex Mesoscale assays detecting IFNα2a, IFNβ, IFN*γ*, IFNλ1, TNFα, IL-1β, IL-6 and CXCL10 (K15067L-2, MSD) according to the manufacturer’s instructions.

### IFN Autoantibodies

IFN autoantibodies were measured in serum as previously described (19). Briefly, ELISA plates were coated with 1 µg/mL IFNα (130-093-874, Miltenyi Biotec) or IFNω (BMS304, Invitrogen) overnight at 4°C followed by blocking in 5% skimmed milk. Serum samples were diluted 50x in HPE buffer (M1940, Sanquin) before incubation. Bound autoantibodies were detected with HRP-conjugated goat anti-human IgG, IgA, IgM (GAHu/Ig(Fc/PO, Nordic-MUbio) and HRP substrate, SureBlue KPL (5120-0077, Sera care).

### DNA Isolation and Whole Exome Sequencing

DNA was isolated from EDTA-blood using the QIAamp DNA Blood Mini Kit (Qiagen, 51104). WES was performed as previously described (20). Variants were only kept if minor allele frequency < 0.1%, CADD > 15 and > mutation significance cutoff and located in coding regions or splice sites. Variants were additionally filtered based on functional prediction by SIFT, Polyphen2, MutationTaster, MutationAssessor, FATHMM and FATHMM-MKL and only kept if at least one prediction tool predicted the variant to be damaging. Remaining variants were then filtered based on biological relevance based on database searches and GO terms. Biologically relevant pathways and functions were chosen based on available literature on MIS-C, given that the MIS-A literature is currently very limited. Dysregulated NK cell, CD8+ T cell, and CD4+ T cell responses have been described in MIS-C, as well as prolonged plasmablast responses and presence of autoantibodies (14, 16, 21), thus genes related to adaptive and humoral immune responses were investigated. MIS-C is also characterized by hyperinflammation (14, 22), prompting examination of genes involved in both inflammatory and anti-inflammatory pathways. Importantly, MIS-C has features in common with both Kawasaki disease and hemophagocytic lymphohistiocytosis, and genes associated with these diseases were therefore also investigated. Additionally, as MIS-C occurs after SARS-CoV-2 infection, host genes involved in viral replication, viral restriction, autophagy, and innate immunity, including IFN production, were also investigated. Although MIS-C and severe acute COVID-19 have different presentations, loci which have already been associated with severe COVID-19 (6) were included in the variant filtering. Finally, all remaining variants were categorized according to American College of Medical genetics (ACMG) guidelines using the ACMG classifier in the bioinformatics program Varseq, and only variants classified as “variant of unknown significance (VUS)”, “likely pathogenic” or “pathogenic” were kept.

### Statistics

Experiments were performed once but all stimulations were done in triplicate. Statistics were calculated using Kruskal-Wallis with correction for multiple comparisons using Dunn’s test. *p<0.05, **p<0.01.

## Results

### Cases

**Figure 1:**
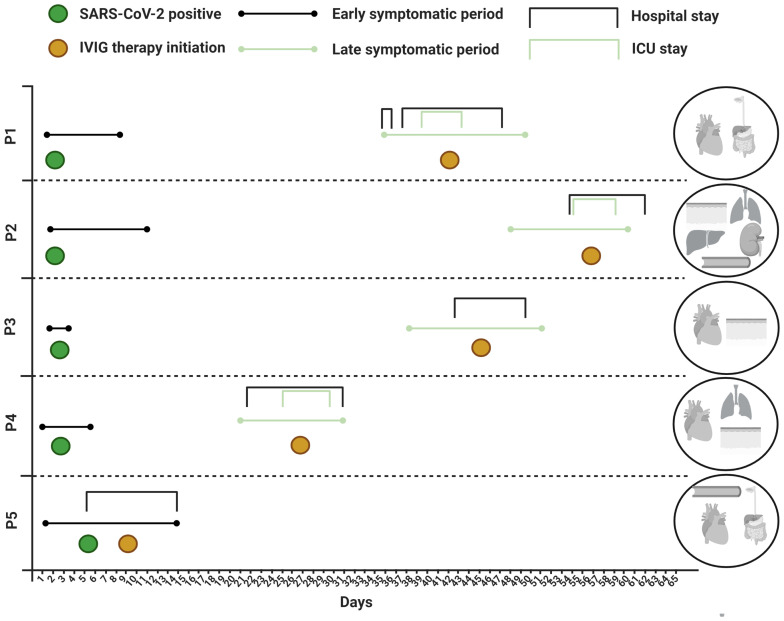
Disease course and organ manifestations. ICU, intensive care unit; IVIG, intravenous immunoglobulin; SARS-CoV-2, severe acute respiratory syndrome coronavirus 2.

P1 was a 19-year-old Brazilian born male. He had an unremarkable medical history except for mild asthma. Four weeks after a mild PCR-confirmed SARS-CoV-2 infection (**Figure 1**), he presented with severe headache and fever accompanied by a sore throat, vomiting and loose stools. Meningitis was initially suspected but ruled out after a lumbar puncture, and he was discharged from hospital the same day. Two days later he was re-admitted with persistent fever and severe abdominal pain, diarrhea and elevated biochemical markers of inflammation and slightly elevated liver enzymes. Severe gastroenteritis was suspected, and ceftriaxone was initiated. On the third day of hospitalization, P1 developed severe acute hypotension and hypoxia. Myocarditis was suspected and a transthoracic echocardiography (TTE) revealed global hypokinesia with an estimated left ventricular ejection fraction (LVEF) of ~15% consistent with the clinical picture of cardiogenic shock. The patient was transferred to a cardiac intensive care unit (ICU) where inotropic therapy was initiated. Ceftriaxone was changed to piperacillin/tazobactam and ciprofloxacin. Intravenous immunoglobulin (IVIG) was initiated on the suspicion of MIS. An endomyocardial biopsy (EMB) revealed lymphocytic infiltration of the myocardium with endotheliitis and minor necrosis (**Figure S1**). The patient experienced significant improvement after supportive therapy and fever resolved within 12-24 hours after IVIG treatment. He was hospitalized for another five days before discharge and is currently under full recovery. LVEF at follow-up was mildly reduced (50%). An overview of the clinical work-up is supplied as online supplementary material (**Table S1**). All reverse transcriptase polymerase chain reaction (PCR) of respiratory specimens and the EMB were negative for SARS-CoV-2.

P2 was a 33-year-old previously healthy white male. Approximately five weeks prior to admission the patient tested PCR-positive for SARS-CoV-2 with respiratory symptoms, including cough and slight dyspnea not requiring hospitalization. Upon return from a business travel to Cape Town, South Africa, he was admitted to hospital with respiratory and circulatory failure requiring immediate invasive mechanical respiratory support, aggressive fluid resuscitation and vasopressor therapy. Furthermore, a universal rash with target lesions resembling erythema multiforme was observed (**Figure S2**). He had experienced five days of fever and two days of dry cough and dyspnea as well as abdominal discomfort with diarrhea prior to hospitalization. Empirical antibiotic therapy with ceftriaxone and clarithromycin was initiated, and shock reversal therapy with hydrocortisone was added shortly after ICU admittance. He had biochemical signs of inflammation and laboratory evidence of liver injury and renal failure, and a chest X-ray revealed bilateral pulmonary infiltrates. An initial TTE in the emergency department was normal but a follow-up TTE within hours after intubation revealed global hypokinesia (LVEF ~20%), and troponin T and pro-brain-natriuretic peptide (NT-proBNP) levels were elevated. The following day, hemodialysis was initiated due to renal failure with hyperkalemia together with IVIG therapy on the suspicion of MIS-A. Extensive microbiological evaluation did not reveal an underlying etiology (**Table S2**) and a transesophageal echo (TEE) showed no signs of infective endocarditis. One oropharyngeal swab, out of four respiratory specimens, came out PCR-positive for SARS-CoV-2, and the patient additionally had a positive SARS-CoV-2 antibody test. He improved rapidly following IVIG initiation and required a total of six days of hospitalization before discharge and has now fully recovered.

P3 was a 23-year-old white female admitted seven weeks after a positive SARS-CoV-2 PCR test. Her initial symptoms were mild lethargy lasting for a few days. A week prior to admission she developed myalgia, irritation of the throat, cough, expectoration, retrosternal chest pain and high fever. She had also experienced conjunctival injections and chapped lips, which were not evident at admission. On suspicion of pneumonia she was prescribed penicillin and ciprofloxacin, both of which were discontinued after a few doses. The biochemical work up included elevated inflammatory markers, cardiac enzymes and NT-proBNP (**Table S3**). Chest x-ray was normal, and no causative pathogens were identified. Electrocardiography revealed sinus tachycardia with T-wave inversion across all precordial leads. No arrythmias were detected during telemetry. MIS-A was suspected, and IVIG, methylprednisolone and acetylsalicylic acid was initiated three days after admission. TTE was normal but cardiac magnetic resonance (MR) imaging on the second day of admission was consistent with myocarditis. Clinical improvement and biochemical normalization ensued shortly after immunomodulatory therapy and the patient was discharged at day seven.

P4 was a 50-year-old Chilean-born male. Apart from familial hypercholesterolemia, he was previously healthy. Three weeks prior to admission he experienced flu-like symptoms and tested PCR-positive for SARS-CoV-2. He quickly recovered, but eighteen days later he was hospitalized with fever, headache, sore throat, aching muscles and joints as well as abdominal pain. He had laboratory signs of inflammation, and intravenous fluids and empirical piperacillin-tazobactam were administered. On the second day of hospitalization, swollen lymph nodes on the left side of the neck and a widespread rash was noted. Fiberoptic laryngoscopy was normal and ultrasound of the neck revealed enlarged lymph nodes with suspicion of an abscess adjacent to the left tonsil. Contrast-enhanced CT of the neck, chest and abdomen confirmed the presence of enlarged cervical lymph nodes and enlarged left tonsil and suggested a possible retropharyngeal abscess on the left side of the neck. Retropharyngeal and cutaneous surgical explorations were performed, without signs of abscesses. The left tonsil was removed, and the histology was without significant pathology. Intravenous meropenem, clindamycin and metronidazole were initiated, and piperacillin-tazobactam was discontinued. On the fifth day of hospitalization, he still had fever and rising inflammatory parameters and was admitted to the ICU due to respiratory distress. Contrast-enhanced chest-CT revealed bilateral consolidations without signs of pulmonary embolism and a TTE showed mild depressed LV systolic function (LVEF 40-45%) with no valvular lesions or pericardial effusion. NT-proBNP levels increased from 922 pmol/l to 5530 pmol/l from day five to seven. The differential diagnosis consisted of Lemierre’s syndrome but repeat CT venography showed no signs of thrombophlebitis and extensive microbiologic evaluations did not reveal an underlying microbial cause (**Table S3**). On the eighth day of hospitalization, the patient still had fever and MIS-A was suspected. Hydrocortisone and IVIG were administered and broad-spectrum antibiotics were upheld. The following day, the fever subsided, the inflammatory parameters decreased, and the patient was discharged from hospital three days later and recovered fully.

P5 was a previously healthy 38-year-old woman born in Lebanon. Six days prior to admission she developed fever, sore throat and diarrhea. Upon arrival to hospital she appeared dehydrated and was febrile (39.5°C) with biochemical signs of inflammation (**Table S5**). Throat swab was SARS-CoV-2 PCR positive (SARS-CoV-2 tests were not performed in the weeks prior to admission). During the first day of admission the patient developed hypotension (blood pressure 80/60 mmHg). Chest X-ray was normal, but TNT was elevated to maximum of 2953 ng/l with a NT-proBNP of 6753 ng/l and a TTE revealed an LVEF of 40%. The patient was rehydrated with fluids and started piperacillin/tazobactam. Microbiological analyses did not identify another causative pathogen (**Table S5**). On day 3, MIS-A was suspected and IVIG was initiated. The following day the fever subsided, and the patient reported significant improvement. Over the next three days the blood pressure and inflammatory makers normalized. A repeat TTE on day eight showed normal LVEF. The patient was discharged on day 9 and fully recovered.

### Immunological Evaluations

#### SARS-CoV-2 Specific Cytokines and IFN Responses *In Vitro* and IFN Autoantibodies

**Figure 2:**
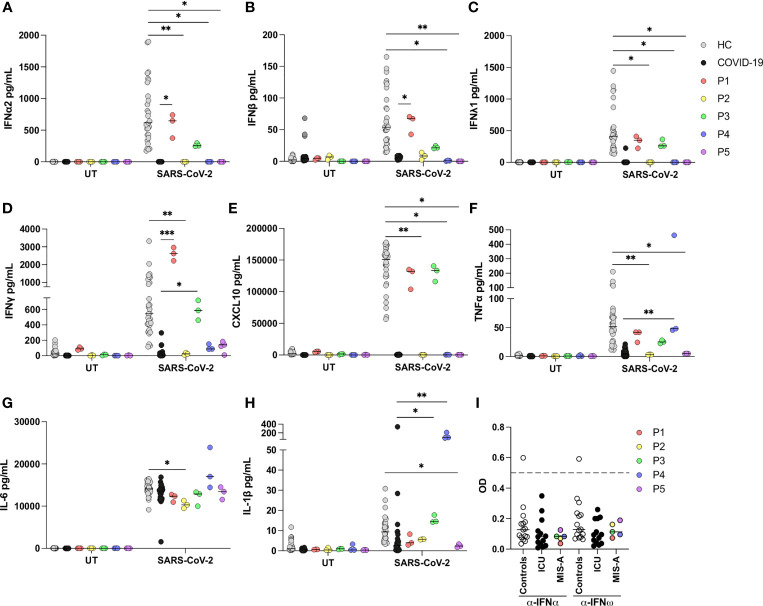
Antiviral and inflammatory responses of patient PBMCs in response to SARS-CoV-2 infection. **(A–H)** Patient (P1-P5), healthy controls (HC) (n=5) and COVID-19 intensive care unit (ICU) patient (COVID-19) (n=9) peripheral blood mononuclear cells were left untreated (UT) or infected with SARS-CoV-2 at a multiplicity of infection of 0.5 for 24 h. Induction of IFNs and proinflammatory cytokines was measured in supernatants by U-plex mesoscale technology. Data are pooled from two independent experiments, but the experiment was performed only once for each patient due to limited patient material. All stimulations were done in triplicate in the individual experiments. **(I)** Autoantibodies against IFNα and IFNω measured in serum from patients, healthy controls (n=15) and COVID-19 ICU patients (n=13) by ELISA, y-axis depicts blank-corrected OD450-630. Values above 0.5 (dashed line) are considered positive. Statistical differences were calculated using Kruskal-Wallis with correction for multiple comparisons by Dunn’s test. *p < 0.05, **p < 0.01, ***p < 0.001. Each line, showing the result of a statistical test, compares one patient with the HC or the COVID-19 ICU group. Only comparisons between patient and controls, which are statistically different, are indicated on the graph.

P2, P4, and P5 all exhibited significantly impaired production of type I IFN (IFNα and IFNβ) and type III IFN (IFNλ) in response to SARS-CoV-2 infection compared to healthy controls (HC), whereas responses from MIS-A patients and ICU patients with severe/critical COVID-19 were comparable (**Figures 2A–C**). Type II IFN (IFN*γ*) was only significantly decreased in PBMCs from P2 compared to HC, but increased in P1 and P3 compared to COVID-19 patients (**Figure 2D**). Production of the IFN stimulated gene (ISG) CXCL10 followed the pattern as observed for type I and III IFNs with almost completely abolished responses in PBMCs from P2, P4 and P5 compared to HC (**Figure 2E**). Production of proinflammatory cytokines was less affected. TNFα was decreased in P2 and P5 compared to HC, but increased in P4 compared to COVID-19 ICU patients. Interleukin (IL)-6 and IL-1β responses were normal in all patients, except P2 and P5, respectively, where these markers were reduced compared to HCs (**Figures 2F–H**). P3 and P4 exhibited increased IL-1β production compared to COVID-19 patients (**Figures 2F–H**). None of the patients had autoantibodies levels against IFNα or IFNω above 0.5, which is considered the threshold for positive samples (**Figure 2I**). Unexpectedly, one of the 15 healthy controls had measurable IFN autoantibodies.

### Identification of Potentially Disease-Causing Genetic Variants by Whole Exome Sequencing

To investigate the presence of gene variants that may have contributed to development of MIS-A WES was performed on DNA obtained form P1-P5. This resulted in a final list of 11 missense and one nonsense variant distributed among the five patients (**Table 1**). These 12 variants could be divided into four subgroups according to the biological function of the affected gene, including i) autophagy (*LGALS8, TECPR1*), ii) Kawasaki disease (*PEAR1, ERAP1*), iii) viral restriction factors (*PLIN3, EXOSC5, RNASE2*), and iv) immune responses (*ERAP1, SIGLEC15, GAB2, GOLGA4, SNX3*).

**Table 1:** Genetic variants identified in P1-P5 -by whole exome sequencing.

	Gene	Transcript	Transcript variant	Protein variant	CADD Score	CADD MSC	Frequency gnomAD exomes	dbSNP ID	SIFT	Polyphen2	Mutation taster	Mutation Assessor	FATHMM	FATHMM MKL	ACMG Classification
**P1**	*GAB2*	NM_080491.2	c.856G>A	p.Glu286Lys	25.1	3.313	1.59082e-05	rs199954922	Damaging	Possibly damaging	Damaging	Predicted functional (medium)	Tolerated	Damaging	VUS/Weak Pathogenic
	*PLIN3*	NM_005817.4	c.356C>T	p.Ala119Val	22.7	3.313	9.15408e-05	rs146293739	Damaging	Benign	Damaging	Predicted functional (medium)	Tolerated	Damaging	VUS/Weak Benign
	*SIGLEC15*	NM_213602.2	c.503C>G	p.Pro168Arg	24.8	3.313	0.000247915	rs200427190	Damaging	Probably damaging	Damaging	Predicted functional (medium)	Damaging	Damaging	VUS/Weak Pathogenic
**P2**	*GOLGA4*	NM_001172713.1	c.302C>T	p.Pro101Leu	27.2	3.313	4.37553e-05	rs373167384	Damaging	Probably damaging	Damaging	Predicted non-functional (low)	Tolerated	Damaging	VUS/Weak Pathogenic
	*FMNL1*	NM_005892.3	c.2597C>T	p.Ala866Val	21.8	3.313	1.99807e-05	rs201789328	Tolerated	Benign	Damaging	Predicted non-functional (neutral)	Tolerated	Tolerated	VUS/Conflicting
**P3**	*LGALS8*	NM_006499.4	c.326T>G	p.Val109Gly	25.8	3.313	2.38791e-05	rs753660901	Damaging	Probably damaging	Damaging	Predicted functional (high)	Tolerated	Damaging	VUS/Weak Pathogenic
**P4**	*EXOSC5*	NM_020158.3	c.535G>A	p.Ala179Thr	24.9	3.313	4.94947e-05	rs151051277	Tolerated	Benign	Damaging	Predicted non-functional (low)	Tolerated	Damaging	VUS/Weak Pathogenic
	*RNASE2*	NM_002934.2	c.187C>T	p.Arg63*	26.6	3.313	0.000469413	rs141786181	NA	NA	Damaging	NA	NA	Tolerated	VUS
	*TECPR1*	NM_015395.2	c.77A>C	p.Glu26Ala	22.8	3.313	0.000200234	rs369593510	Tolerated	Benign	Damaging	Predicted non-functional (low)	Tolerated	Damaging	VUS
**P5**	*ERAP1*	NM_001040458.1	c.982A>G	p.Arg328Gly	23.5	3.313	0.00015119	rs371320592	Damaging	Probably damaging	Damaging	Predicted functional (medium)	Tolerated	Tolerated	VUS
	*PEAR1*	NM_001080471.1	c.625G>A	p.Ala209Thr	19.7	3.313	3.19137e-05	rs372909968	Damaging	Benign	Tolerated	Predicted non-functional (low)	Tolerated	Tolerated	VUS/Weak Pathogenic
	*SNX3*	NM_003795.5	c.175A>G	p.Ile59Val	22	5.3	0.000134676	rs371370665	Tolerated	Benign	Damaging	Predicted non-functional (neutral)	Tolerated	Damaging	VUS

Samples were filtered based on frequency, deleteriousness and biological relevance. Only variants with minor allele frequency < 0.01%, CADD > 15, (and > CADD MSC), predicted damaging by at least one other prediction tool (SIFT, Polyphen2, Mutation Taster, Mutation Assessor, FATHMM or FATHMM MKL) and evaluated as being biologically relevant were kept. All variants were heterozygous and missense, except in the case of RNASE2 which was a premature STOP. CADD, combined annotation dependent depletion; MSC, mutation significance cutoff; NA, not available; VUS, variant of unknown significance.

*Stop codon.

SARS-CoV-2 inhibits autophagic flux, suggesting that autophagy is an antiviral mechanism against SARS-CoV-2 (23). Two of our patients, P3 and P4, harbored variants in genes, *LGALS8* and *TECPR1*, which are involved in autophagy. Galectin-8 (encoded by *LGALS8*) senses membrane damage induced by infection, both bacterial and viral, and subsequently targets the pathogens for autophagy (24, 25). TECPR1 interacts with ATG5 and is necessary for autophagy of bacterial pathogens (26). Recently, it was also demonstrated that TECPR1 in addition to ATG5 also interacts with LC3C and promotes fusion of autophagosomes with lysosomes (27).

Two variants detected in genes linked to Kawasaki disease, were detected in P5. A different *PEAR1* variant than the one identified in the present study has previously been associated with risk of coronary artery aneurism in Kawasaki disease (28), whereas *ERAP1* was identified as a novel susceptibility locus for Kawasaki disease in a genome-wide association study (29). In addition, *ERAP1* encodes an aminopeptidase important for processing of peptide fragments for MHC presentation, various SNPs in ERAP1 has been associated with infectious diseases, including viral infections (30).

One variant in P1 and two variants in P4 were detected in genes, *PLIN3, EXOSC5* and *RNASE2*, which are viral restriction factors. Perilipin 3 (encoded by *PLIN3*), also known as TIP47, has a proviral function in HIV-1 and vaccinia virus infections, whereas it functions as a viral restriction factor in Sendai virus and human parainfluenza 3 virus infections (31, 32). EXOSC5 interacts with HBV RNA leading to RNA degradation and thus viral restriction (33). RNASE2, also known as EDN, has antiviral activity against extracellular respiratory syncytial virus particles and against HIV *in vitro* (34, 35). Additionally, RNASE2 has also been shown to cleave RNA from various pathogens enabling recognition by the endosomal RNA sensor TLR8 and hereby induce activation of antimicrobial responses (36).

Finally, several variants, identified in P1, P2 and P5, were detected in genes involved in various parts of the immune system, primarily the adaptive immune response. SIGLEC15 inhibits T cells proliferation and activation, and SNPs in this gene has been associated with *Candida albicans* infections and pulmonary tuberculosis (37). GAB2 regulates Th2 and mast cell immune responses (38, 39). GOLGA4, also known as p230, regulates expression of HLA class I molecules (40). *FMNL1* encodes a protein involved in remodeling of the cytoskeleton upon interaction between T cells and B cells or antigen presenting cells during formation of immune synapses (41). Finally, SNX3 is a regulator of phagocytosis in immune cells (42).

## Discussion

The patients shared characteristic features of multisystem inflammatory syndrome (10) although organ involvement and the time course of disease varied slightly. They had no prior history of immunodeficiency, autoinflammatory or autoimmune disease. All had prolonged fever, marked signs of inflammation and evidence of myocardial dysfunction in combination with other organ manifestations including severe hypotension (four patients), gastrointestinal symptoms (four patients), skin involvement (two patients), lymphadenopathy of the neck (one patient), coagulopathy (one patient) and hepatic injury with renal failure (one patient). All patients had evidence of COVID-19 with a positive PCR-test at admission or within weeks before admission, and no other obvious cause of inflammation. Despite severe illness with organ dysfunction, requiring treatment in the ICU for three patients, all five patients recovered rapidly after initiation of IVIG with or without corticosteroids.

Several hypotheses have been presented to explain the systemic inflammatory response and organ involvement in MIS-A/C patients, including antibody enhancement, immune complex-mediated immunopathology, superantigen-mediated immune activation, interferonopathy and others (14, 16, 17). A recent study performing cytokine profiling in plasma from nine MIS-C patients identified signatures of inflammation (IL-18 and IL-6), lymphocytic and myeloid chemotaxis and activation (CCL3, CCL4, and CDCP1), and mucosal immune dysregulation (IL-17A, CCL20, and CCL28) (15). Whether MIS-A patients have a similar cytokine response to MIS-C patients is unknown. Moreover, several questions regarding specific antiviral immune responses are unanswered. We infected patient PBMCs with SARS-CoV-2 and observed significantly impaired type I and III IFN responses as well as reduced expression of the ISG CXCL10. A recent study similarly demonstrated very low levels of type I IFNs and ISGs in MIS-C patients, although this was measured in serum and not PBMC culture supernatants (43). In contrast, induction of proinflammatory cytokines revealed a tendency towards increased TNFα production and IL-6 production similar to controls. MIS-A patient PBMC responses to SARS-CoV-2 were largely similar to those of PBMCs from ICU patients with severe COVID-19, however IFN*γ*, TNFα; and IL-1β responses tended to be higher in MIS-A PBMCs although not significantly across all cases. Collectively, these findings may indicate that the high level of proinflammatory mediators previously detected in plasma may not be directly induced by SARS-CoV-2 itself. Rather the primary mechanism at work may be that a secondary post-infectious autoinflammatory or autoimmune reaction, possibly related to the persistent presence of viral antigens, and/or the ability of such viral antigens to trigger an exaggerated immune response with autoimmunity after an acute SARS-CoV2 infection.

The precise mechanism whereby impaired type I and III IFN responses may contribute to disease pathogenesis remains uncertain. Impairment of primarily type I IFN has been shown to be associated with severe COVID-19 pneumonia. It may be hypothesized that an early impaired SARS-CoV-2 specific IFN response, alongside unaffected production of inflammatory mediators, may predispose to MIS-C/A. However, this hypothesis is partly contradicted by the characteristic findings of mild or even asymptomatic SARS-CoV-2 infection in individuals subsequently developing MIS-C/A, as was also the case in three out of five of the patients described here. Two functionally similar but mechanistically independent mechanisms related to the type I IFN response, i.e. neutralizing autoantibodies against type I IFNs (19) and inborn errors of TLR3- and IRF7-dependent type I IFN immunity (6), have been demonstrated to be associated with severe COVID-19. To our knowledge, the presence of autoantibodies in the serum from MIS-C/A patients has not previously been studied. Type I IFN autoantibodies were not detected in any of the five patients described here, although the limited number of cases presented here precludes any specific conclusion to be drawn concerning the presence and a potential pathogenic role of IFN autoantibodies in the pathogenesis of MIS-A.

All five patients (P1-P5) received immunomodulatory treatment(s), including IVIG and for some corticosteroids, which may have impacted on PBMC responsiveness. Samples from P2, P4 and P5 were obtained while the patients received either IVIG together with steroid treatment (P2), steroid tapering (P4), or IVIG alone (P5). Thus, it cannot be excluded that pharmacologically induced immunomodulation may have contributed to the suppressed antiviral IFN response. However, previous studies on cytokine production in response to mitogen-stimulated PBMCs in the presence of IVIG showed no change in several cytokines, including TNF-α and IFN-γ (44), and the normal capacity to induce IL-6 in the present study demonstrates the integrity of the cells and these pathways. Additional investigations of antiviral immune responses in larger MIS-A patient series, ideally with serial measurement, and with comparison to plasma cytokine profiling, are needed to confirm these findings.

To explore whether inborn errors of IFN immunity, or other potential genetic disease-causing variants, could be identified, we performed WES. The knowledge on potential contributing genetic factors in MIS-C/A pathogenesis is very limited. A recent case report described development of MIS-C following SARS-CoV-2 infection in one patient with haploinsufficiency of SOCS1 (a negative regulator of IFN signaling) (45). WES analyses did not reveal any variants in SOCS1 or other IFN-related genes but identified 12 rare potentially disease-causing variants with diverse functions in autophagy, Kawasaki disease, viral restriction, and immune responses. Autophagy was recently found to be inhibited by SARS-CoV-2 (23) suggesting that autophagy may have antiviral functions in SARS-CoV-2 infection. The finding of genetic variants in autophagy genes in P3 and P4 (*LGALS8* and *TECPR1*) and viral restriction factors in P1 and P4 (*PLIN3, EXOSC5*, and *RNASE2*) suggests that defective autophagy-mediated inhibition of viral replication might play a role in development of MIS-A. Only P5 had variants in genes (*PEAR1, ERAP1*) previously linked to Kawasaki disease. A *PEAR1* polymorphism has previously been associated with coronary artery aneurism in Kawasaki disease (28) and *ERAP1* was identified as a susceptibility locus for Kawasaki disease in a Chinese population (29). However, further studies are mandated to functionally evaluate and validate these findings in additional patients.

In conclusion, we provided a clinical, immunological and genetic description of five MIS-A patients. Striking differences in innate antiviral responses, particularly affecting type I and type III IFN responses, were present in patient cells after SARS-CoV-2 infection *in vitro*, which may suggest defects and dysregulation in immunity in these patients. Finally, the identification of several rare potentially disease-causing gene variants may serve as a catalogue for generating hypotheses for future studies addressing molecular genetics and immunological factors associated with susceptibility and pathogenesis of MIS-A.

## Data Availability Statement

The original contributions presented in the study are included in the article/**Supplementary Material**. Further inquiries can be directed to the corresponding author.

## Ethics Statement

The studies involving human participants were reviewed and approved by Danish National and Regional Committee on Health Ethics (1-10-72-80-20 and H‐2–2009‐131). The patients/participants provided their written informed consent to participate in this study. Written informed consent was obtained from the individual(s) for the publication of any potentially identifiable images or data included in this article.

## Author Contributions

AR, AEH, and THM conceived the idea and planned the study. AR, AEH, RP, FG, CR, RE, and MS cared for patients, collected patient material and consent and provided clinical data. SJ performed whole exome sequencing and bioinformatics analysis and performed experiments and analyses on patient cells and serum. SJ and THM interpreted data. AR, SJ and THM wrote the first draft of the article. All authors contributed to the article and approved the submitted version.

## Funding

This work was supported by the NOVO Nordisk Research Foundation (NNF20OC0064890, NNF20OC0063436 and NNF21OC0067157).

## Conflict of Interest

The authors declare that the research was conducted in the absence of any commercial or financial relationships that could be construed as a potential conflict of interest.

## Publisher’s Note

All claims expressed in this article are solely those of the authors and do not necessarily represent those of their affiliated organizations, or those of the publisher, the editors and the reviewers. Any product that may be evaluated in this article, or claim that may be made by its manufacturer, is not guaranteed or endorsed by the publisher.

## Abbreviations

CXCL10, C-X-C motif chemokine ligand 10; EMB, Endomyocardial biopsy; IFN, Interferon; IRF, Interferon regulatory factor; IVIG, Intravenous immunoglobulin; MIS-A, Multisystem inflammatory syndrome in adults; MIS-A, Multisystem inflammatory syndrome in children; LVEF, Left ventricular ejection fraction; PBMC, Peripheral blood mononuclear cells; NTproBNP, Pro-brain-natriuretic peptide; SOCS1, Suppressor of cytokine signaling 1; TLR, Toll-like receptor; TTE, Transthoracic echocardiography; WES, Whole exome sequencing.
