# Supplementary material for: Host Genetics and Antiviral Immune Responses in Adult Patients With Multisystem Inflammatory Syndrome
Source: Front Immunol. 2021 Aug 31;12:718744. doi: 10.3389/fimmu.2021.718744 (PMC8439578; doi:10.3389/fimmu.2021.718744)
Supplement: Supplementary file 1 [file DataSheet_1.docx]

**ONLINE DATA SUPPLEMENT**

## Host genetics and antiviral immune responses in adult patients with multisystem inflammatory syndrome

## Andreas Ronit, MD, PhD; Sofie E. Jørgensen, MSc, PhD; Casper Roed, MD, PhD; Robert Eriksson, MD, PhD; Ulrik W. Iepsen, MD, PhD; Ronni R. Plovsing, MD, PhD; Merete Storgaard, MD, PhD; Finn Gustafsson, MD, PhD, DMSc; PhD; Ann-Brit E. Hansen, MD, PhD; Trine H. Mogensen^2^, MD, PhD, DMSc

**Contents**

[**Table S1. Laboratory results, imaging and microbiological investigations of patient 1. 3**](#_Toc78033877)

[**Table S2. Laboratory results, imaging and microbiological investigations of patient 2 4**](#_Toc78033878)

[**Table S3. Laboratory results, imaging and microbiological investigations of patient 3. 5**](#_Toc78033879)

[**Table S4. Laboratory results, imaging and microbiological investigations of patient 4. 6**](#_Toc78033880)

[**Table S5. Laboratory results, imaging and microbiological investigations of patient 5 7**](#_Toc78033881)

[**Figure S1. Endomyocardial biopsy of P1 8**](#_Toc78033882)

[**Figure S2. Clinical photo of P2 9**](#_Toc78033883)

| **P1** | Day 1* | Day 2 | Day 3 | Day 4 | Day 5 | | Day 6 | Day 7 |
| --- | --- | --- | --- | --- | --- | --- | --- | --- |
| Hemoglobin level, g/l (8.3-10.5) | 7.6 | 7.5 | 7.3 | 6.8 | 6.4 | | 7.2 | 6.9 |
| Leukocyte count, x 10^9^ cells/L (3.5-8.8) | 5.2 | 6.0 | 7.1 | 7.1 | 10.5 | | 7.2 | 8.7 |
| Platelet count, x 10^9^ cells/L (145-390) | 100 | 100 | 100 | 103 | 117 | | - | - |
| Plasma creatinine, µmol/L (60-105) | 190 | 180 | 300 | 270 | 289 | | 263 | 99 |
| C-reactive protein level, mg/L (<10) | 140 | - | 190 | 180 | 300 | | 270 | 290 |
| Plasma lacate, mmol/L (0.7-2.1) | 0.6 | - |  | 2.7 | 1.9 | | 2.7 | 2 |
| D-dimer, FEU/L (<0.5) | - | - | 1.6 | 5.0 | - | | - | - |
| Alanine aminotransferase, U/L | 83 | 78 | 72 | 53 | 66 | | 59 | 84 |
| Brain-type natriuretic peptide, pmol/L (<10.1) | **-** | - | **-** | 1720 | - | | - | 388 |
| Troponin T, ng/L (<14) |  |  |  | 150 |  | |  | 80 |
| Imaging (not described in text) |  | Abdominal ultrasound: Splenomegaly; chest x-ray: normal | Lung ultrasound: Bilateral multiple B-lines. | Chest x-ray: cardiomegaly, partial LLL atelectasis |  | |  |  |
| HIV serology, hepatitis B/C serology, influenza A/B and RSV PCR, SARS-CoV-2 PCR, CMV/EBV serology, blood and urine cultures, stool PCR (bacteria, viruses, parasites), CSF microscopy and culture. | | | | | | All investigations negative. | | |

Table S1. Laboratory results, imaging and microbiological investigations of patient 1. Abbreviations: Abbreviations: CMV, Cytomegalovirus; CSF, cerebrospinal fluid; EBV, Epstein-Barr virus; LLL, left lower lobe; PCR, polymerase chain reaction; RSV, respiratory syncytial virus; SARS-CoV-2, severe acute respiratory syndrome coronavirus 2. *Day 1 refers to the first day of the second hospital admission.

| **P2** | Day 1 | Day 2 | Day 3 | Day 4 | Day 5 | | Day 6 | Day 7 |
| --- | --- | --- | --- | --- | --- | --- | --- | --- |
| Hemoglobin level, g/l (8.3-10.5) | 9.4 | 8.3 | 7.3 | 7.1 | 7.5 | | 8.4 | 7.8 |
| Leukocyte count, x 10^9^ cells/L (3.5-8.8) | 19.1 | 16.9 | 14.8 | 12.9 | 10.5 | | 9.9 | 8.9 |
| Platelet count, x 10^9^ cells/L (145-390) | 282 | 307 | 290 | 172 | 197 | | 249 | 290 |
| Plasma creatinine, µmol/L (60-105) | 135 | 160 | 140 | 122 | 137 | | 92 | 81 |
| C-reactive protein, mg/L (<10) | 260 | 230 | 200 | 130 | 63 | | 33 | 16 |
| Ferritin level, µg/L (12-300) | - | >2000* | - | - | >2000* | | - | - |
| Plasma lactate, mmol/L (0.7-2.1) | 2.0 | 2.2 | 3.0 | 2.7 | 1.9 | | 1.2 | - |
| D-dimer, FEU/L (<0.5) | 2.7 | 9.9 | 12 | 19 | 52 | | - | - |
| Alanine aminotransferase, U/L (10-70) | 199 | 2110 | 5080 | 3180 | 1970 | | 1330 | 1220 |
| Brain-type natriuretic peptide, pmol/L (<10.1) | - | 1830 | - | - | - | | - | - |
| Troponin T, ng/L (<14) | - | 326-483-380 | - | - | 63 | | - | - |
| Imaging (not described in text) | Chest x-ray: Bilateral interstitial infiltrates | Chest x-ray: Bilateral interstitial infiltrates | Chest x-ray: Bilateral interstitial infiltrates | Abdominal ultrasound: small amount of ascites | Chest CT: LUL segmental lung embolus. | |  |  |
| HIV RNA/serology, hepatitis A/B/C/E serology, influenza A/B/RSV PCR, FilmArray® respiratory PCR panel, *M. pneumoniae/L.pneumophila/C. pneumonia/C. psittaci* PCR, LUAT/PUAT, *P. jirovecii* PCR, SARS-CoV-2 PCR/serology, CMV/EBV serology, chlamydia/gonorrhea urine PCR, blood and urine cultures, stool PCR (bacteria, viruses, parasites), malaria microscopy/antigen test, leptospira urine PCR, *Rickettsia typhi/rickettsii* antibodies, rickettsia species PCR. | | | | | | All investigations negative, except positive SARS-CoV-2 antibodies and one positive SARS-COV-2 PCR oropharyngeal swab. | | |

Table S2. Laboratory results, imaging and microbiological investigations of patient 2**.** Abbreviations: CMV, Cytomegalovirus; EBV, Epstein-Barr virus; LUAT, *L. pneumophila* urinary antigen test; LUL, left upper lobe; PCR, polymerase chain reaction; PUAT, *S. pneumonia* urinary antigen test; RNA, ribonucleic acid; RSV, respiratory syncytial virus; SARS-CoV-2, severe acute respiratory syndrome coronavirus 2. *Not quantified.

| **P3** | Day 1 | Day 2 | Day 3 | Day 4 | Day 5 | | Day 6 | Day 7 |
| --- | --- | --- | --- | --- | --- | --- | --- | --- |
| Hemoglobin level, g/l (7.3-9.5) | 8,0 | 7,9 | 7,9 | 7,6 | 6,7 | | 7,0 | 6,9 |
| Leukocyte count, x 10^9^ cells/L (3.5-8.8) | 7,2 | 10,9 | 19,3 | 21,1 | 15,3 | | 14,4 | 10,9 |
| Platelet count, x 10^9^ cells/L (145-390) | 198 | 186 | 246 | 230 | 246 | | 339 | 354 |
| Plasma creatinine, µmol/L (50-90) | 67 | 67 | 68 | 94 | 56 | | 64 | 53 |
| C-reactive protein, mg/L (<10) | 299 | 376 | 410 | 231 | 239 | | 113 | 60 |
| Ferritin, µg/L (12-300) | - | - | 734 | 1180 | 1400 | | - | - |
| Alanine aminotransferase, U/L (10.45) | 24 | 27 | 27 | 30 | 39 | | - | - |
| Brain-type natriuretic peptide, pmol/L (<14) | - | - | 713 | - | - | | - | - |
| Troponin I, ng/l (<45) | - | - | 672 | - | - | | - | - |
| Troponin T, ng/l (<14) | - | - | 216 | 69 | 94 | | 114 |  |
| Imaging (not described in text) |  | Chest x-ray: Normal |  | MRI heart: Non-dilated LV, small inferoposterior area late gadolinium positive, LVEF of 55% | | | | |
| HIV serology, SARS-CoV-2 PCR/serology, blood, urine and expectorate cultures, *M. pneumoniae/L. pneumophila/C. pneumoniae/C. psittaci* PCR, SARS-CoV-2 IgM/IgG antibodies. | | | | | | All investigations negative including negative SARS-CoV-2 antobodies. | | |

Table S3. Laboratory results, imaging and microbiological investigations of patient 3. Abbreviations: CMV, Cytomegalovirus; EBV, Epstein-Barr virus; LUAT, *L. pneumophila* urinary antigen test; LUL, left upper lobe; LVEF, left ventricular ejection fraction; PCR, polymerase chain reaction; PUAT, *S. pneumonia* urinary antigen test; RNA, ribonucleic acid; RSV, respiratory syncytial virus; SARS-CoV-2, severe acute respiratory syndrome coronavirus 2.

| **P4** | Day 1 | Day 2 | Day 3 | Day 4 | Day 5 | Day 6 | Day 7 | Day 8 | | Day 9 | Day 10 | Day 11 |
| --- | --- | --- | --- | --- | --- | --- | --- | --- | --- | --- | --- | --- |
| Hemoglobin, g/l (8.3-10.5) | 9.2 | 9.3 | 8.3 | 8.4 | 7.8 | 7.8 | 7.7 | 7.0 | | 6.6 | 7.0 | 7.1 |
| Leukocyte count, x 10^9^ cells/L (3.5-8.8) | 13.4 | 13.4 | 12.0 | 13.3 | 15.5 | 21.4 | 22.5 | 22.3 | | 15.5 | 16.1 | 16.8 |
| Platelet count, x 10^9^ cells/L (145-390) | 219 | 188 | 160 | 181 | 181 | 163 | 160 | 231 | | 214 | 266 | 370 |
| Plasma creatinine, µmol/L (60-105) | 91 | 108 | 82 | 98 | 83 | 80 | 89 | 75 | | 59 | 72 | 73 |
| C-reactive protein, mg/L (<10) | 60 | 86 | 110 | 152 | 162 | 261 | 328 | 284 | | 180 | 67 | 44 |
| Ferritin, µg/L (12-300) | -- | -- | -- | -- | 941 | -- | 1190 | 1460 | | -- | 2860 | -- |
| Plasma lactate, mmol/L (0.7-2.1) | 1.6 | 0.7 | 0.6 | 1.3 | 1.4 | 1.2 | 2.3 | 1.4 | | 1.3 | 1 | -- |
| D-dimer, nmol/L (<0.5) | -- | 0.42 | -- | -- | 3.9 | -- | 5.1 | -- | | -- | -- | -- |
| Alanine aminotransferase, U/L (10-70) | 54 | 53 | -- | 51 | 65 | 73 | 53 | 61 | | 121 | 199 | 224 |
| Troponin T, ng/l (<14) |  |  |  |  | 165 |  |  |  | |  |  |  |
| Brain-type natriuretic peptide, pmol/L (<106) | -- | -- | -- | -- | 922 | -- | 5530 | -- | | -- | -- | -- |
| Blood, urine and expectorate cultures, SARS-CoV-2 and influenza virus type A and B PCR, *M. pneumoniae/L. pneumophila/C. pneumoniae/C. psittaci* PCR, SARS-CoV-2 PCR/serology and HIV serology, LUAT/PUAT, assays for antinuclear antibodies, anti-double-stranded DNA and rheumatoid factor. | | | | | | | | | All investigations negative except positive SARS-CoV-2 antibodies. | | | |

Table S4. Laboratory results, imaging and microbiological investigations of patient 4. Abbreviations: LUAT, *L. pneumophila* urinary antigen test; PCR, polymerase chain reaction; PUAT, *S. pneumonia* urinary antigen test; RSV, respiratory syncytial virus; SARS-CoV-2, severe acute respiratory syndrome coronavirus 2

| **P5** | Day 1 | Day 2 | Day 3 | | Day 4 | Day 5 | Day 6 | Day 7 |
| --- | --- | --- | --- | --- | --- | --- | --- | --- |
| Hemoglobin, g/l (8.3-10.5) | 7.0 | 5.4 | 4.4 | | 4.6 | 5.0 | 5.8 | 5.5 |
| Leukocyte count, x 10^9^ cells/L (3.5-10.5) | 9.5 | 6.3 | 7.9 | | 9.2 | 9.4 | 9.7 | 13..6 |
| Platelet count, x 10^9^ cells/L (145-350) | 224 | 157 | 231 | | 356 | 490 | 663 | 686 |
| Plasma creatinine, µmol/L (60-105) | 59 | 51 | 45 | | 57 | 49 | 53 | 50 |
| C-reactive protein, mg/L (<8) | 299 | 284 | 286 | | 271 | 155 | 86 | 47 |
| Ferritin, µg/L (22-355) | 233 | 225 | 231 | | 356 | 282 | 179 | 115 |
| Plasma lactate, mmol/L (0.5-2.5) | 1.2 | - | - | | - | - | - | - |
| D-dimer, nmol/L (<0.5) | - | 2.1 | 2.0 | | 2.9 | 4.2 | 4.9 | 3.7 |
| Alanine aminotransferase, U/L (10-70) | 16 | 16 | 38 | | 144 | 135 | 117 | 125 |
| Brain-type natriuretic peptide, ng/L (<300) | - | 6753 | - | | - | 5198 | - | 2220 |
| Troponin (HS), ng/l (<47) | 2253 | 998 | 300 | | 13 | 41 | - | - |
| HIV serology, hepatitis B and C serology. Blood and feces culture. | | | | All investigations negative, except one positive SARS-CoV-2 oropharyngeal swab. | | | | |

Table S5. Laboratory results, imaging and microbiological investigations of patient 5**.** Abbreviations: PCR, polymerase chain reaction; PUAT, *S. pneumonia* urinary antigen test; RNA, ribonucleic acid; RSV, respiratory syncytial virus; SARS-CoV-2, severe acute respiratory syndrome coronavirus 2. *Not quantified.


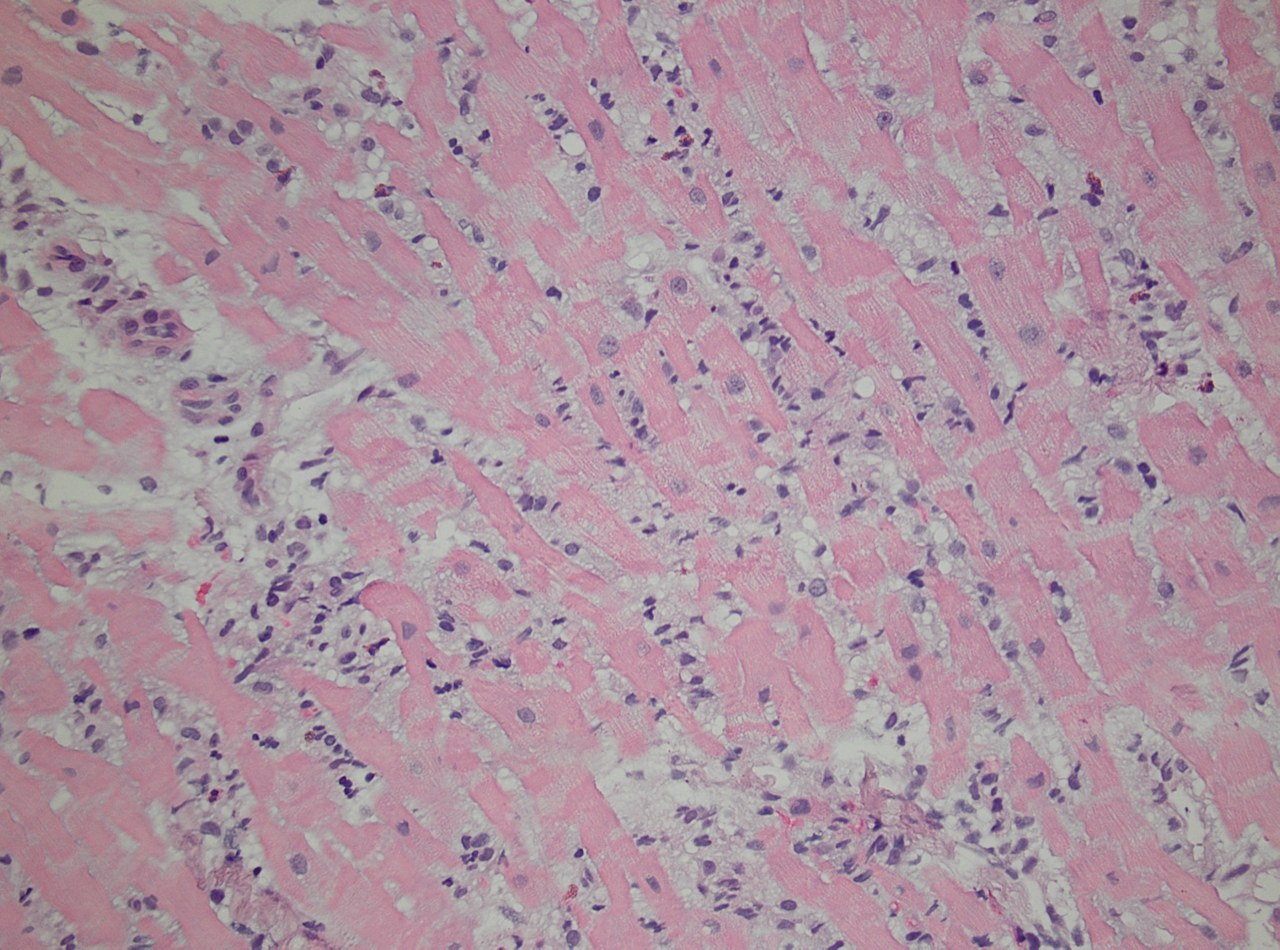


Figure S1. Endomyocardial biopsy of P1**.** Hematoxylin-eosin stain (x40) shows active endomyocarditis with associated capillaritis and vasculitis. Inflammatory cells are dominated by lymphocytes and macrophages, but a few eosinophils and neutrophils can also be identified. Subsequent immunohistochemistry confirmed lymphohistiocytic inflammation with mostly CD3+ T-lymphocytes and CD68+ macrophages. No pathological deposition of iron, glycogen or amyloid.


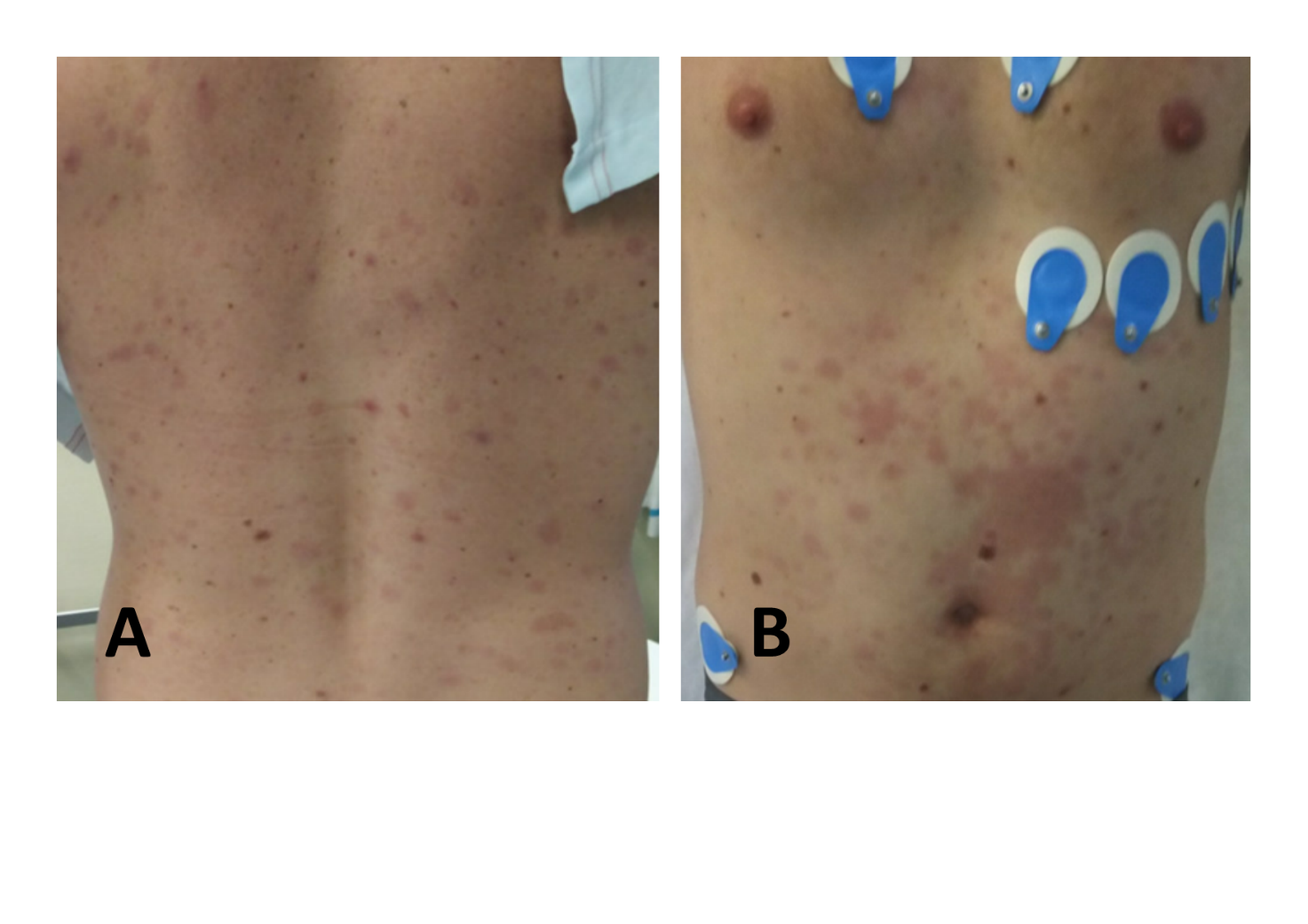


Figure S2. Clinical photo of P2**.** Shows the patient from a) the back and b) the front on the day of admission. He presented with a universal macular to slightly raised erythematous rash sparing the face and palms, soles and mucous membranes. The rash disappeared within days.
